# A Microplate Growth Inhibition Assay for Screening Bacteriocins against *Listeria monocytogenes* to Differentiate Their Mode-of-Action

**DOI:** 10.3390/biom5021178

**Published:** 2015-06-11

**Authors:** Paul Priyesh Vijayakumar, Peter M. Muriana

**Affiliations:** 1 Department of Animal Science, Oklahoma State University, Monroe Street, Stillwater, OK 74078, USA; E-Mail: paul.v@uky.edu; 2 Robert M. Kerr Food & Agricultural Products Centre, Oklahoma State University, 109 FAPC Building, Monroe Street, Stillwater, OK 74078-6055, USA; E-Mail: peter.muriana@okstate.edu

**Keywords:** bacteriocins, *Listeria monocytogenes*, microplate assay, inhibition, biopreservatives

## Abstract

Lactic acid bacteria (LAB) have historically been used in food fermentations to preserve foods and are generally-recognized-as-safe (GRAS) by the FDA for use as food ingredients. In addition to lactic acid; some strains also produce bacteriocins that have been proposed for use as food preservatives. In this study we examined the inhibition of *Listeria monocytogenes* 39-2 by neutralized and non-neutralized bacteriocin preparations (Bac^+^ preps) produced by *Lactobacillus curvatus* FS47; *Lb. curvatus* Beef3; *Pediococcus acidilactici* Bac3; *Lactococcus lactis* FLS1; *Enterococcus faecium* FS56-1; and *Enterococcus thailandicus* FS92. Activity differences between non-neutralized and neutralized Bac^+^ preps in agar spot assays could not readily be attributed to acid because a bacteriocin-negative control strain was not inhibitory to *Listeria* in these assays. When neutralized and non-neutralized Bac^+^ preps were used in microplate growth inhibition assays against *L. monocytogenes* 39-2 we observed some differences attributed to acid inhibition. A microplate growth inhibition assay was used to compare inhibitory reactions of wild-type and bacteriocin-resistant variants of *L. monocytogenes* to differentiate bacteriocins with different modes-of-action (MOA) whereby curvaticins FS47 and Beef3, and pediocin Bac3 were categorized to be in MOA1; enterocins FS92 and FS56-1 in MOA2; and lacticin FLS1 in MOA3. The microplate bacteriocin MOA assay establishes a platform to evaluate the best combination of bacteriocin preparations for use in food applications as biopreservatives against *L. monocytogenes*.

## 1. Introduction

*Listeria monocytogenes* is a human pathogen linked to foodborne illness outbreaks involving dairy, poultry, and ready-to-eat (RTE) meat products. It has been a recurrent threat to RTE deli meats and hotdogs, frequently due to post-process contamination [1]. Conventional antimicrobials (potassium lactate and sodium diacetate) approved by the U.S. FDA have been effective in inhibiting this pathogen and are considered the comparative standard of the industry for antimicrobial interventions in RTE meats. Consumers are generally concerned about possible health effects from the presence of chemical additives in foods and as a result, they are often drawn to natural and “fresher” foods with no added chemical preservatives. This perception, coupled with the increasing demand for minimally-processed foods with long shelf life and convenience, together with recurring problems with *Listeria* in RTE foods, has stimulated research interest in finding natural, but effective, preservatives. Bacteriocins produced by LAB are natural preservatives that can possibly fulfill these requirements. Lactic acid bacteria (LAB) are generally-recognized-as-safe (GRAS) by the FDA and the bacteria themselves, or their cultured byproducts, can be freely used in foods as food ingredients. Some strains of LAB are also known for the production of bacteriocins (*i.e.*, antimicrobial peptides). Bacteriocins have been proposed for use as biopreservatives, either as direct food additives (*i.e.*, nisin), as pasteurized/condensed cultured food ingredients (*i.e.*, cultured milk or whey), or by using the cultures themselves to ferment products or be used as protective inoculants [2,3,4,5].

Optical density measurement is a straight forward approach for monitoring bacterial growth and determining the inhibitory effects of antimicrobials from plants, spices, and foods and can be readily accommodated by microplate readers. Rufián-Henares and Morales [6] used a microplate reader method to evaluate the antimicrobial properties of melanoidins which are generally present in coffee, beer, and sweet wine against *Escherichia coli* and *Staphylococcus aureus*. Assays using 96-well microplate systems have been proposed to quantify bacteriocin activity in cell free extracts [7,8]. Additionally, Turcotte *et al.* [9] developed a 1-day turbidometric assay for quantification of purified nisin Z and pediocin PA-1 activity in fermented media to evaluate the relationship between indicator strain growth and bacteriocin concentration.

The objectives of the current study were to use a turbidometric-based microplate assay to assess the inhibition of *L. monocytogenes* 39-2 by six bacteriocin preparations. The goal was to develop an *in vitro* assay to distinguish bacteriocins of different modes-of-action (MOA) for their future use in a mixed-MOA motif as natural preservatives in food applications.

## 2. Results and Discussion

### 2.1. Heat Tolerance of Bacteriocins

**Figure 1:**
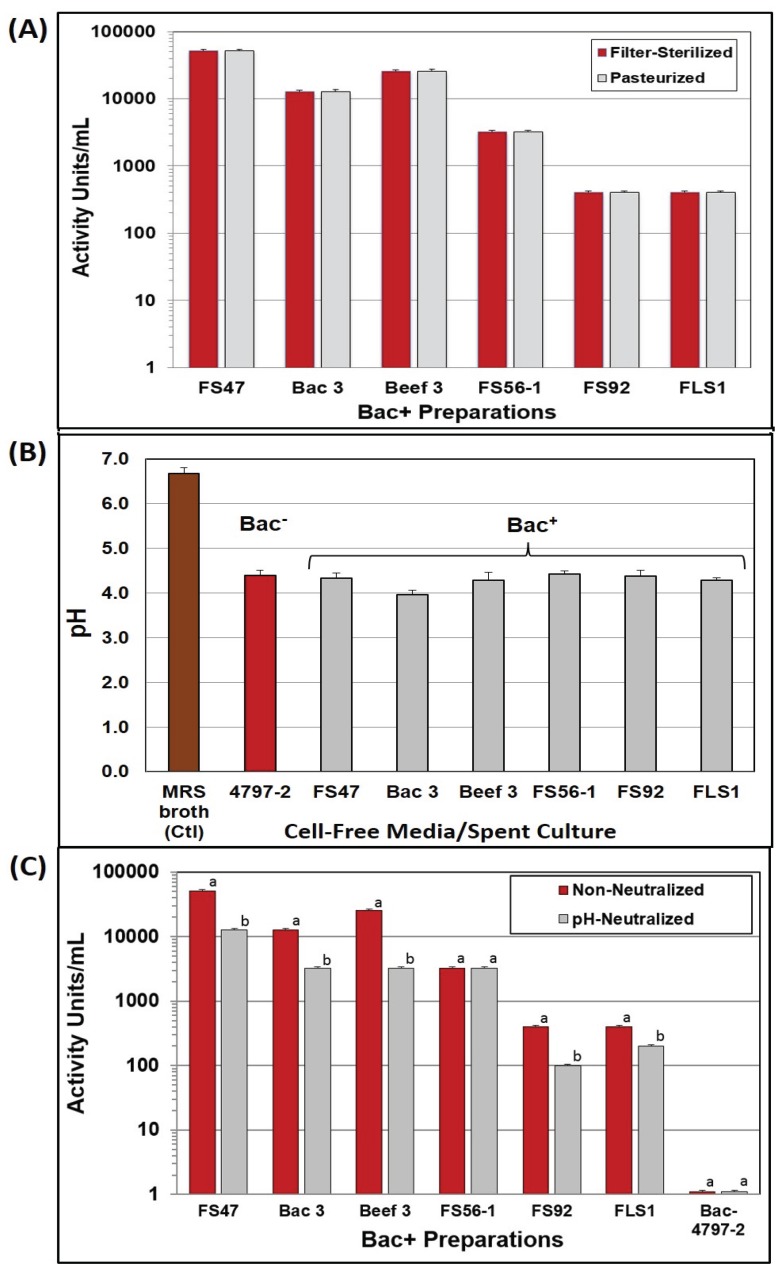
Treatment of culture supernatants produced by *Lb. delbrueckii* 4797-2 (Bac^−^) and Bac^+^ strains: *Lb. curvatus* FS47, *P. acidilactici* Bac3, *Lb. curvatus* Beef3, *En. faecium* FS56-1, *En. thailandicus* FS92, and *Lc. lactis* FLS1. (**A**) Comparison of bacteriocin activity after filter sterilization or heat pasteurization; (**B**) The pH of MRS broth media before inoculation and after overnight growth of various strains used in this study; (**C**) Bacteriocin activity titers of cell-free supernatants against *L. monocytogenes* 39-2, before and after neutralization. Treatments with the same culture supernatant that have different lower case letters are significantly different (*p* < 0.05); treatments with the same lowercase letter are not significantly different (*p* > 0.05). No differences were found in duplicate replications of samples in (**A**) and (**C**).

Bacteriocin culture preparations were examined for heat resistance, both as a potential replacement for filter sterilization and as an indication that bacteriocin preparations would survive applications in heated foods. We observed no loss in activity when centrifuged/heat treated bacteriocin preparations were compared to filter-sterilized preparations, nor any differences between duplicate samples (Figure 1A). In subsequent heating trials, we increased the temperature to 85 °C for 15–20 min, because of the use of larger samples, obtaining similar results. The ability to survive high heat treatment without loss of activity demonstrates that these bacteriocins may be added to foods that will be heated and still retain activity. The bacteriocins used in this study demonstrate thermal stability under conditions simulating pasteurization and confirm heat resistance observed in other bacteriocins [10].

### 2.2. Bacteriocin Activity at Acid vs. Neutral pH

Nearly all of the Bac^−^ and Bac^+^ cultures lowered the pH of MRS broth (~pH 6.7) down to ~pH 4.3 (Figure 1B) allowing the use of a standardized neutralization regimen on a fixed volume of cell-free supernatant culture. A pH-effect was observed on bacteriocin activity titers of cell-free supernatants obtained before, and after, pH neutralization in which five of six showed reduced activity after neutralization (Figure 1C). This might not seem unusual since LAB can produce both lactic acid and bacteriocins, and perhaps neutralization eliminated inhibition due to lactic acid while leaving only bacteriocin activity. However, no inhibitory activity was observed on *Listeria* indicator lawns from non-neutralized supernatant from the Bac^−^ strain, *Lb. delbrueckii* 4797-2 (Figure 1C) and therefore it is not clear why neutralized Bac^+^ preps should show changes in inhibitory activity.

**Figure 2:**
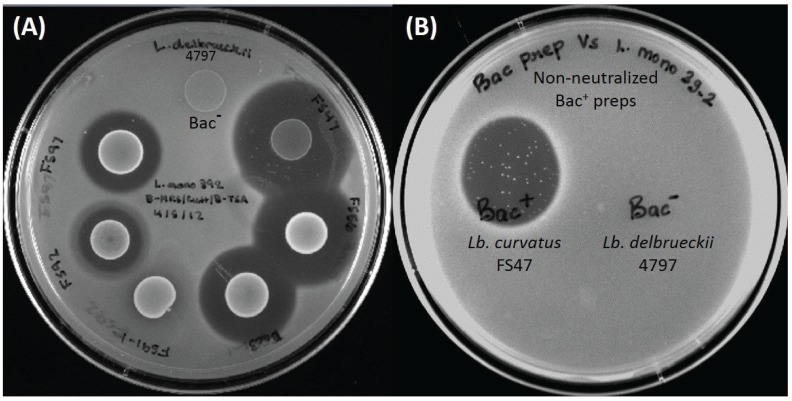
(**A**) Inhibition of *L. monocytogenes* 39-2 by overlay of prior spotted and incubated culture spots (“deferred antagonism”). Spotted cultures, clockwise from top: *Lb. delbrueckii* 4797-2 (Bac^−^ control), *Lb. curvatus* FS47, *En. faecium* FS56-1, *Pediococcus acidilactici* Bac3, *Lactococcus lactis* FS91-1, *En. thailandicus* FS92, *En. faecium* FS97-2; (**B**) Inhibition of *L. monocytogenes* indicator lawn spotted with non-neutralized cell free spent broth from Bac^+^ (*Lb. curvatus* FS47) and Bac^−^ (*Lb. delbrueckii* 4797-2) strains (“concurrent antagonism”). The presence of “spontaneous resistant” indicator colonies can be clearly observed in the curvaticin FS47 inhibition zone.

Traditional concepts of organic acid inhibition in foods maintains that organic acids, such as lactic acid, are mostly inhibitory to bacteria when the pH of the food medium in which the bacteria are suspended is at or below the pK_a_ of the acid (*i.e.*, pH 3.86 for lactic acid). That is when the majority of the lactic acid molecules are nondissociated ([Ac^−^H^+^]) and able to diffuse into bacterial cells because of their net-neutral charge. When the pH is above the pK_a_ of the acid, it is mostly in the dissociated form ([Ac^−^] + [H^+^]) and the charge on the lactate anion prevents its entry into cells (or after the nondissociated form gains entry into cells, it dissociates to the toxic anion form in the neutral pH of the bacterial cytoplasm). In the current study the pH of the Bac^−^ spent broth (and five of six Bac^+^ preps) was at ~pH 4.37 and above the pK_a_ of lactic acid and showed no inhibitory activity on agar when applied to lawns of *L. monocytogenes* 39-2 indicator cells (Figure 1C and Figure 2).

A possible explanation of the pH-related phenomena observed in this study is that the net charge of the bacteriocin is also involved in adsorption to cell surfaces of Gram-positive bacteria. Most bacteriocins have higher antimicrobial activity at pH 5 or lower, compared to their activity at physiological pH [11,12,13]. At pH 6 or above, bacteriocin molecules adsorb to the surface of bacteriocin producer cells and other Gram-positive bacteria, in effect, titering them out of solution [7,12,14,15,16,17]. In general, at low pH, bacteriocins do not adsorb well to bacterial cells, so more is available for inhibitory functionality and increased antibacterial activity is observed. When culture supernatants are neutralized, activity due to acid is eliminated, but bacteriocin activity is also diminished because of this absorption phenomenon.

### 2.3. Wild-Type L. monocytogenes 39-2 (R0) and Bacteriocin-Resistant Variants (R1, R2)

**Figure 3:**
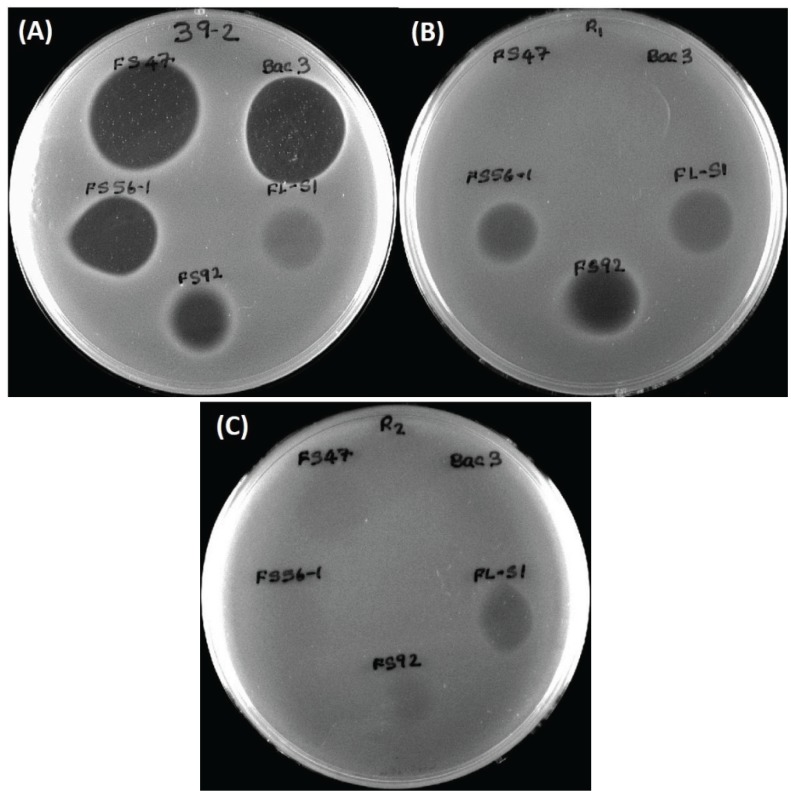
(**A**) *L. monocytogenes* 39-2 (R0) spotted with cell free supernatants from five Bac^+^ strains: *Lb. curvatus* FS47, *P. acidilactici* Bac3, *Lactococcus lactis* FLS1, *En. thailandicus* FS92, *En. faecium* FS56-1; (**B**) *L. monocytogenes* 39-2 (R1) spotted with the same five Bac^+^ supernatants; (**C**) *L. monocytogenes* 39-2 (R2) spotted with the same five Bac^+^ supernatants.

A bacteriocin-resistant variant of the wild-type *L. monocytogenes* 39-2 (“R0”; *i.e.*, not resistant) obtained against curvaticin FS47 was designated “R1” (*i.e.*, resistant #1). A comparison of inhibitory bacteriocin spots on lawns of *L. monocytogenes* R0 and R1 demonstrated that the spontaneous resistance against *Lb. curvatus* bacteriocin FS47 gave cross-resistance to *P. acidilactici* bacteriocin Bac3 (Figure 3) and *Lb. curvatus* bacteriocin Beef3 [18]. Some bacteriocins no longer inhibited *L. monocytogenes 39-2* (R1) while others were still inhibitory to it (Figure 3B). The different bacteriocins whose activity were no longer inhibitory to the R1 Bac^R^ variant (*i.e.*, FS47, Bac3, and Beef3) were classified as having the same MOA while those that were still inhibitory to R1 (*i.e.*, FS56-1, FS92) were considered to possess another MOA. This process was again repeated by isolating additional enterocin FS56-1 Bac^R^ mutants against R1 that possessed accumulated bacteriocin resistances, now designated “R2” (Figure 3C). Activity against R2 identified a bacteriocin (*i.e.*, FLS1) of still another MOA (Figure 3C). The wild type *L. monocytogenes* (R0) and its two consecutively-derived Bac^R^ variants (R1, R2) were used as indicator organisms to screen for new Bac^+^ isolates as well as screen bacteriocin preparations in microplate inhibition assays [19]. 

### 2.4. Microplate Growth Inhibition Assays Using Wild-Type L. monocytogenes 39-2 (R0)

**Figure 4:**
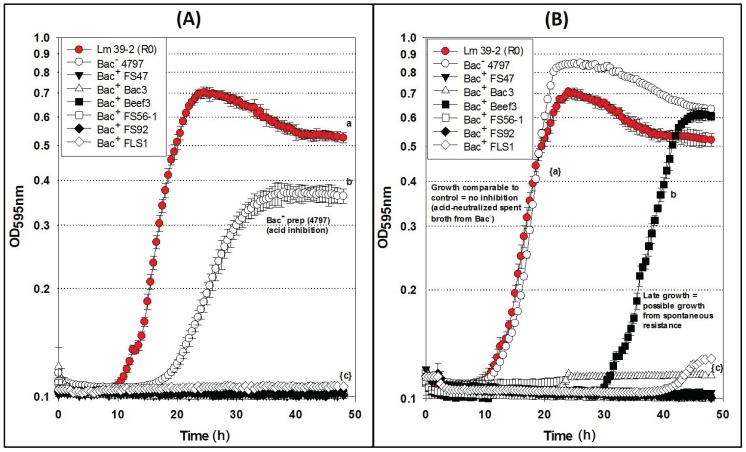
Microplate growth inhibition assays showing the activity of *Listeria monocytogenes* 39-2 (R0) treated with culture supernatants from: None (R0 Cont.), *Lb. delbrueckii* 4797-2 (Bac^−^), *Lb. curvatus* FS47, *P. acidilactici* Bac3, *Lb. curvatus* Beef3, *En. faecium* FS56-1, *En. thailandicus* FS92, and *Lc. lactis* FLS1. (**A**) Microplate assay using non-neutralized culture supernatants; (**B**) Microplate assay using neutralized culture supernatants. Data points represent the means of triplicate replications and error bars represent the standard deviations from the means (error bars were not used for all curves in order to prevent clutter). Treatments with different lowercase letters are significantly different (*p* < 0.05); letters in brackets are for entire group of graph lines.

Differences were observed with Bac^−^ preps in microplate growth inhibition assays in which *L. monocytogenes* 39-2 (R0) was treated with neutralized or non-neutralized cell-free culture supernatants (Figure 4) that were not observed during agar spot activity titer assays (Figure 1C). When non-neutralized cell-free supernatants of the Bac^−^ control strain (*Lb. delbrueckii* 4797-2) were added to *L. monocytogenes* R0, some inhibition was observed whereby R0 did not grow as well as in the Bac^−^ control assay while all the Bac^+^ preparations showed baseline-level inhibition (Figure 4A). However, when neutralized cell-free supernatants of the Bac^−^ 4797-2 strain were added to R0, it grew equally well and without any significant difference from the R0 control (Figure 4B). Trials with the neutralized preps also showed late recovery of *L. monocytogenes* R0 treated with the Beef3 Bac^+^ prep (Figure 4B) suggesting possible outgrowth of spontaneous resistant variants which is consistent with observations in the agar spot assay for FS47 (Figure 2B) and other bacteriocins (Figure 3A). The baseline-level inhibition of *L. monocytogenes* by bacteriocin Beef3 observed with non-neutralized preparations (Figure 4A) could have been due to acid-assisted inhibition that was removed by neutralization; such differences are likely only noticeable with microplate growth inhibition data, offering real-time growth curve comparisons, that would otherwise appear as similar fully-grown endpoints on agar surface spot assays.

### 2.5. Microplate Assay of Bacteriocin Preparations vs. L. monocytogenes 39-2 (R1)

**Figure 5:**
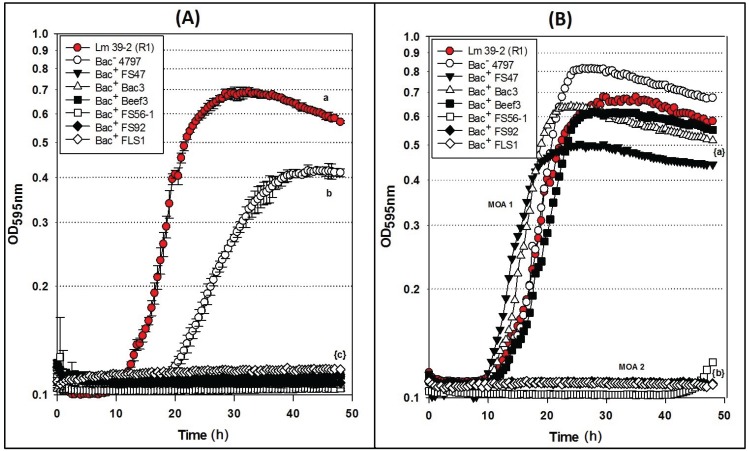
Microplate growth inhibition assays showing the activity of *Listeria monocytogenes* 39-2 (R1) treated with culture supernatants from: None (R1 Cont.), *Lb. delbrueckii* 4797-2 (Bac^−^), *Lb. curvatus* FS47, *P. acidilactici* Bac3, *Lb. curvatus* Beef3, *En. faecium* FS56-1, *En. thailandicus* FS92, and *Lc. lactis* FLS1. (**A**) Microplate assay using non-neutralized culture supernatants; (**B**) Microplate assay using neutralized culture supernatants. Data points represent the means of triplicate replications and error bars represent the standard deviations from the means (error bars were not used for all curves in order to prevent clutter). Treatments with different lowercase letters are significantly different (*p* < 0.05); letters in brackets are for entire group of graph lines.

Microplate inhibition assays were also performed with the *L. monocytogenes* 39-2 R1 Bac^R^ variant (Figure 3B) and growth inhibition assays were again evaluated with non-neutralized and neutralized cell-free culture supernatants (Figure 5). As observed previously, the R1 strain with non-neutralized supernatants from the Bac^−^ control strain showed intermediate inhibition (Figure 5A) whereby the neutralized supernatants from the Bac^−^ control showed no inhibition (Figure 5B). This time, three of six neutralized Bac^+^ preps showed no inhibition of R1 due to the bacteriocin resistance incurred by strain R1 (Figure 5B). All of the bacteriocins that are no longer inhibitory to *L. monocytogenes* 39-2 (R1) are presumed to have the same or highly similar MOA while those bacteriocins that were still inhibitory to R1 were considered to have a different MOA. The data obtained in microplate growth assays with *L. monocytogenes* 39-2 (R1) suggests that curvaticin FS47, curvaticin Beef3, and pediocin Bac3 belong to the same mode of action, MOA1.

### 2.6. Microplate Assay of Bacteriocin Preparations vs. L. monocytogenes 39-2 (R2)

**Figure 6:**
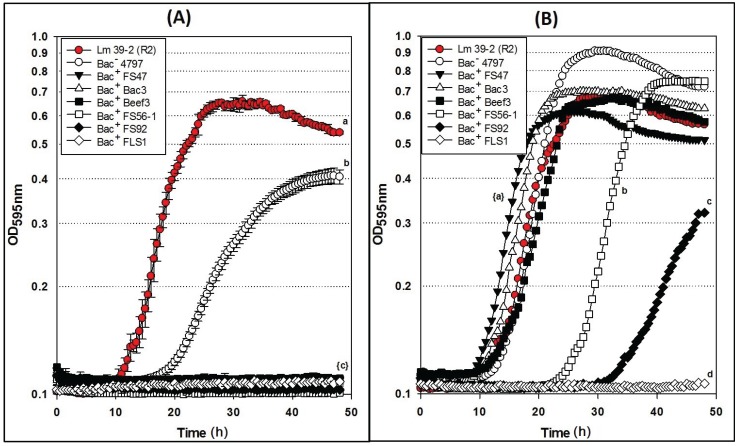
Microplate growth inhibition assays showing the activity of *Listeria monocytogenes* 39-2 (R2) treated with culture supernatants from: None (R2 Cont.), *Lb. delbrueckii* 4797-2 (Bac^−^), *Lb. curvatus* FS47, *P. acidilactici* Bac3, *Lb. curvatus* Beef3, *En. faecium* FS56-1, *En. thailandicus* FS92, and *Lc. lactis* FLS1. (**A**) Microplate assay using non-neutralized culture supernatants; (**B**) Microplate assay using neutralized culture supernatants. Data points represent the means of triplicate replications and error bars represent the standard deviations from the means (error bars were not used for all curves in order to prevent clutter). Treatments with different lowercase letters are significantly different (*p* < 0.05); letters in brackets are for entire group of graph lines.

Similar to the results of the inhibition assays against *L. monocytogenes* 39-2 R0 and R1 performed with non-neutralized culture supernatants, we observed full growth of *L. monocytogenes* 39-2 (R2), partial inhibition of R2 by the Bac^−^ supernatants, and full inhibition from the Bac^+^ supernatants (Figure 6A). When the neutralized cell-free supernatants were added, the R2 Bac^R^ variant showed the same resistance to bacteriocins FS47, Bac3, and Beef3 as R1, but less sensitivity to enterocins FS92 and FS56-1, from which R2 was selectively derived (Figure 6B). The remaining Bac^+^ prep (FLS1) still showed baseline-level inhibition of R2 (Figure 6B). From these data, we suggest that enterocins FS92 and FS56-1 belong to a second mode of action, MOA2, while lacticin FLS1 belongs to a third, MOA3.

These data support that the microplate inhibition assay is more sensitive to acid than the agar spot assays, presumably because of the scale of inhibitory assay applied whereby only 5–10 μL of supernatant is applied as a spot on the agar surface allowing the agar layer below the spot to buffer the acid. In the microplate assay, half of the liquid added to the wells is culture supernatant and background lactic acid may have more influence than in the agar spot assays. The microplate assay allows the observation of discernible inhibition during growth using turbidity data over time. Growth curves of neutralized preparations that appear delayed are likely inhibited to some degree, and may represent unique MOAs that are not readily discernible by the agar plate assay. It is also noteworthy that some bacteriocinogenic strains, including FS56-1 and FS92, have been shown to possess more than one bacteriocin [19,20]. It is reasonable to assume that in such situations, if one bacteriocin is rendered insensitive by a bacteriocin-resistance indicator, but not the other, it is possible that a reduced zone size may be observed in spot on lawn assays (Figure 3B) or intermediate results in microplate growth assays (Figure 6B). Further testing is needed to confirm these hypotheses. Although baseline-level inhibition is always ideal and preferable, bacteriocins that show inhibitory activity at levels between full baseline inhibition and uninhibited growth may still have merit in food applications, especially when using mixtures of bacteriocins of different MOAs.

**Figure A1:**
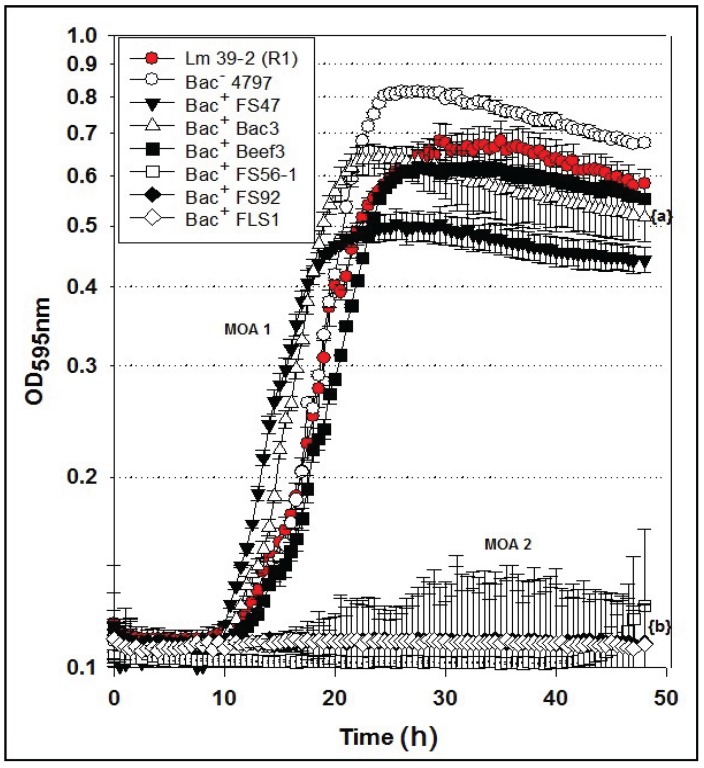
(Figure 5B with error bars) Growth inhibition assay showing the activity of *Listeria monocytogenes* 39-2 (R1) treated with culture supernatants from: None (R1 Cont.), *Lb. delbrueckii* 4797-2 (Bac^−^), *Lb. curvatus* FS47, *P. acidilactici* Bac3, *Lb. curvatus* Beef3, *En. faecium* FS56-1, *En. thailandicus* FS92, and *Lc. lactis* FLS1. Growth inhibition assay using neutralized culture supernatants. Data points represent the means of triplicate replications and error bars represent the standard deviations from the means (error bars were not used for all curves in the main paper to prevent clutter). Treatments with different lowercase letters are significantly different (*p* < 0.05); letters in brackets are for entire group of graph lines.

**Figure A2:**
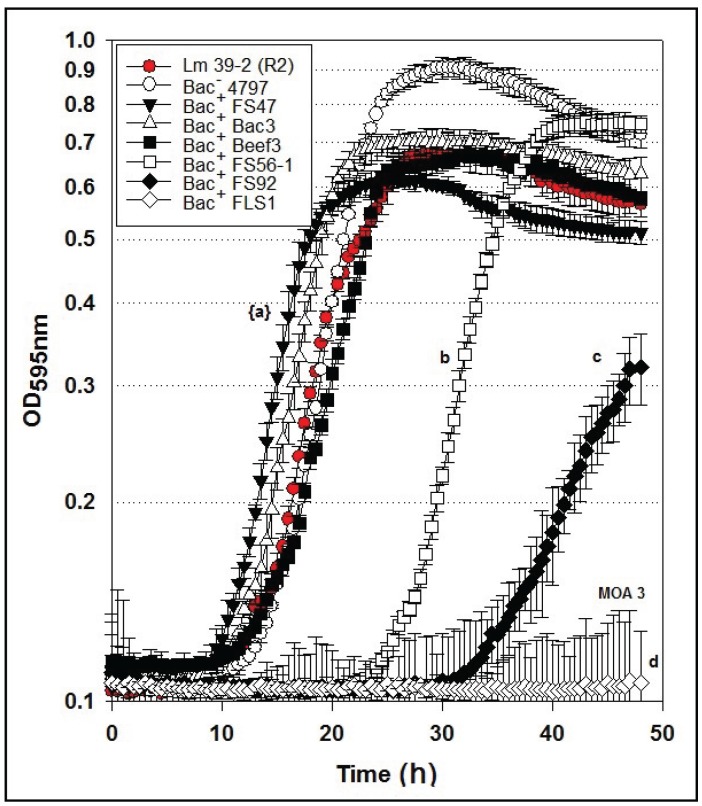
(Figure 6B with error bars) Growth inhibition assay showing the activity of *Listeria monocytogenes* 39-2 (R2) treated with culture supernatants from: None (R2 Cont.), *Lb. delbrueckii* 4797-2 (Bac^−^), *Lb. curvatus* FS47, *P. acidilactici* Bac3, *Lb. curvatus* Beef3, *En. faecium* FS56-1, *En. thailandicus* FS92, and *Lc. lactis* FLS1. Growth inhibition assay using neutralized culture supernatants. Data points represent the means of triplicate replications and error bars represent the standard deviations from the means (error bars were not used for all curves in the main paper to prevent clutter). Treatments with different lowercase letters are significantly different (*p* < 0.05); letters in brackets are for entire group of graph lines.

Another observation in the study was the tailing of most of the growth curves in the microplate assays (Figure 4, Figure 5 and Figure 6). Although the reason was not ascertained, it is likely due to a slow lysis of the bacterial cells as bacteriocins form membrane pores in susceptible cells and/or the result of a change in cellular morphology upon nutrient depletion during extended stationary phase which may have an effect on optical density. Figure 5B with error bars has been published as an Appendix Figure A1; likewise, Figure 6B with error bars has been published as an Appendix Figure A2.

## 3. Experimental Section

### 3.1. Bacterial Cultures, Handling, Growth, and Storage Conditions

Select bacteriocin-producing (Bac^+^) LAB obtained from our culture collection or from retail foods and animal sources [20,21,22] were propagated in de Man, Rogosa, and Sharpe Lactobacilli broth (Difco, Becton-Dickenson Labs, Franklin Lakes, NJ, USA) at 30 °C. Bac^+^ LAB included *Lactobacillus curvatus* FS47 and Beef3, *Pediococcus acidilactici* Bac3, *Lactococcus lactis* FLS1, *Enterococcus faecium* FS56-1 and *En. thailandicus* FS92. All cultures were propagated twice before use. Master cultures were maintained by resuspension of cell pellets in milk-based freezing media (11% non-fat dry milk powder, 1% glucose, 0.2% yeast extract) after centrifugation (8000 rpm, 4 °C, 10 min) and stored frozen. *L. monocytogenes* 39-2 (R0, R1, and R2) were grown in tryptic soy broth (TSB, Difco, Becton-Dickenson Labs). The parent strain, *L. monocytogenes* 39-2 was isolated from packages of retail frankfurters [23], and has been used as part of a 4-strain *L. monocytogenes* “cocktail” for inoculation of RTE meats evaluating meat surface pasteurization [24,25], liquid smoke extracts [26,27] as antimicrobial interventions, and has also been characterized as having moderate biofilm adherence properties by a microplate adherence assay [28]. All (working stock) cultures were held at −20 °C for short term storage or at −80 °C for long term storage.

### 3.2. Bacteriocin Preparations

Cultures of Bac^+^ LAB were propagated overnight (twice) at 30 °C and centrifuged at 8000 rpm (4 °C) for 10 min (Sorvall RC50 Plus, Thermo Fisher Scientific, Waltham, MA, USA). The supernatants (*i.e.*, “Bac^+^ preps”) were filter-sterilized with cellulose acetate syringe filters (25 mm, 0.20 μ pore-size; Nalgene), aliquoted into 1 mL eppendorf tubes for use in experiments, and stored at 4 °C for short term experiments or at −20 °C for longer term storage.

### 3.3. Exclusion of other Inhibitors

The possibility of inhibitory activity produced by other potential inhibitors (bacteriophage, acid inhibition, or hydrogen peroxide) observed among some LAB was eliminated as described previously [19,22].

### 3.4. Neutralization of Culture Supernatants

Culture supernatants were neutralized after centrifugation to remove the bulk of cells (see Section 3.6). An Oakton pH 110 series pH meter (EUTECH instruments, Cole-Parmer, Court Vernon Hills, IL, USA) was used to measure the pH of bacteriocin preparations collected in sterile falcon tubes. The pH electrode was calibrated against pH 4.0 and pH 7.0 buffers and rinsed with deionized water. Cell-free bacteriocin preparations obtained from LAB were neutralized (pH 6.8–7.2) using approximately 80–150 μL of 5 M NaOH per 10 mL volume. After neutralization, the supernatants were filter-sterilized as described earlier.

### 3.5. Bacteriocin Activity Determination by Serial Dilution and Spot-on-Lawn Assay

Filter-sterilized or pasteurized Bac^+^ preps were serially diluted by 2-fold dilutions in 0.1% buffered peptone water (BPW) in 96-well microtiter plates (Becton-Dickenson Labs) whereby the bacteriocin is diluted in half with each successive dilution. Indicator assay plates were made whereby *L. monocytogenes* 39-2 (8-log cfu/mL) was seeded at 1% dilution into soft TSA agar (0.75% agar), and then 5 mL of this seeded soft agar was overlaid onto normal TSA agar plates (1.5% agar). The indicator-seeded assay plates were marked in eight pie-section quadrants and 10 μL of each dilution was surface spotted onto the indicator lawns. Plates were allowed to incubate overnight at 30 °C and the titer end point was taken from highest dilution showing the last visible sign of inhibition. The titer was then designated as activity- or arbitrary-units (AU), and was determined as the reciprocal of the dilution × 100 (because 10 μL represents 1/100^th^ of 1 mL) and reported as AU/mL. 

### 3.6. Heat Pasteurization of Bacteriocin Preparations

LAB cultures were propagated twice at 30 °C and centrifuged at 8000 rpm for 10 min at 4 °C (Sorvall RC 50 Plus). The centrifuged supernatant was transferred to sterile tubes and pasteurized at 80 °C for either 5-min (1 mL portions) suspended in a water-filled heating block. Pasteurized bacteriocins preparations were stored at 4 °C for near term experiments or frozen at −20 °C. Bacteriocin activity was determined as described previously and done in duplicate. Samples of pasteurized bacteriocin preparations were plated onto MRSA plates or inoculated into MRS broth to check the effectiveness of the pasteurization process.

### 3.7. Bacteriocin-Resistant Variants of Wild-Type L. monocytogenes 39-2

During prolonged incubation of inhibition zones during spot-on-lawn assays of bacteriocin preparations against *L. monocytogenes* 39-2, bacteriocin-resistant colonies would appear at frequencies of approximately 10^−5^ to 10^−7^ (Figure 2B and Figure 3A). Bacteriocin-resistant variants of *L. monocytogenes* 39-2 were obtained from select Bac^+^ strains by plating dilutions of overnight culture on TSA that was surface-inoculated with 200–300 μL of cell-free supernatant of Bac^+^ culture (*i.e.*, *Lactobacillus curvatus* FS47). Colonies that arose against the bacteriocin were again streaked again on TSA + Bac^+^ (FS47) to insure isolation of a resistant phenotype. Bacteriocin-resistant (Bac^R^) variants recovered in this manner were tested in spot tests with various cell-free bacteriocin preparations in comparison with the prior strain and was repeated with bacteriocins that still inhibited the Bac^R^ *L. monocytogenes* 39-2 variant. The use of bacteriocin-resistant variants as screening organisms was characterized by Macwana and Muriana [29] as capable of differentiating bacteriocins of different modes-of-action (MOA).

### 3.8. Microplate Turbidometric Growth Inhibition Assays

The microplate inhibition assay used a mixture of indicator organism (*L. monocytogenes* 39-2) and various culture supernatant preparations (Bac^+^, Bac^−^). Sterile MRS broth (not spent broth) was used as a control treatment and spent culture supernatant from bacteriocin-negative (Bac^−^) *Lb. delbrueckii* 4797 was used as a control to assess lactic acid effects. *L. monocytogenes* 39-2 was diluted and inoculated into double-strength TSB broth (~1 × 10^5^ cfu/mL) from which 100 µL was distributed to various wells in a clear 96-well flat bottom microtiter plate (Becton Dickinson). Bacteriocin preparations (100 µL) were added and mixed by aspiration using a multi-channel pipette. Settings for the growth curve/turbidity analysis using a GENios microplate reader (Tecan Inc, Morrisville, NC, USA) were as follows: measurement mode: absorbance; measurement wavelength: 595 nm; number of flashes: 1; temperature range: 33–35 °C; shake duration (orbital normal): 10 s; kinetic interval: 1800 s; unit: optical density (OD); and total measurement time: 48 h. The 96-well plate was sealed with UltraClear film (Axygen Inc., Union City, CA, USA) to prevent evaporation of the liquid and well-to-well contamination. The OD_595_ values obtained were plotted against time and were used to illustrate the antilisterial activity of the bacteriocin preparations against *L. monocytogenes*.

### 3.9. Statistical Analysis

Microplate growth inhibition assays were repeated in triplicate and mean O.D. (595 nm) values were plotted *versus* time. The statistics functions in SigmaPlot 13 (Systat Software, San Jose, CA, USA) was used to perform one-way repeated measures analysis of variance (RM-ANOVA) to determine if significant difference exists between different treatments. For some assays, the Holm-Sidak one way ANOVA method was used to perform pair wise multiple comparisons with level of significance set at 0.05 (*p*-value).

## 4. Conclusions

Bacteriocinogenic LAB and their bacteriocin preparations are promising antimicrobials for application in food systems as effective biopreservatives, especially in RTE meat systems. However, crude screening methods such as agar diffusion assays alone would not be sufficient to forecast their effectiveness in meat systems that are highly complex. In this study we have employed agar diffusion assays (sandwich) only as a primitive approach to screen for new bacteriocinogenic LAB from different food samples. In order to obtain more detailed information on the effectiveness of the bacteriocin preparation on the inhibition of the *L. monocytogenes* 39-2 and to study the interactions of bacteriocins affecting different MOAs, a more reliable growth inhibition assay was developed. The comparison of non-neutralized and pH-neutralized preparations confirmed the participation of acid inhibition against *L. monocytogenes*, while evaluations performed with neutralized preparations allowed preferential evaluation of bacteriocin activities against *L. monocytogenes*. The comparison of wild-type *L. monocytogenes* 39-2 (R0) *vs.* bacteriocin-resistant variants (R1, R2) allowed a preferential selection of bacteriocins that are likely to work better together (*i.e.*, “mixed mode-of-action”) and without confusion of whether activity is due to lactic acid. Although the primary interest was in evaluating only inhibition due to bacteriocin activity during *in vitro* assays, any additional inhibition by lactic acid during actual in-food applications would be an added and appreciable benefit. The use of such *L. monocytogenes* Bac^R^ variants allowed the differentiation of bacteriocins into different MOA’s (MOA1: curvaticins FS47 and Beef3, pediocin Bac3; MOA2: enterocins FS92 and FS56-1; MOA3: lacticin FLS1) from which we may select an efficacious mixture of bacteriocin preparations for application as food preservatives, especially where recurrent problems with *L. monocytogenes* have been observed.
